# *Notes from the Field:* Evaluation of a Perceived Cluster of Plasma Cell Dyscrasias Among Workers at a Natural Gas Company — Illinois, 2014

**DOI:** 10.15585/mmwr.mm6623a5

**Published:** 2017-06-16

**Authors:** Marie A. de Perio, Jayesh Mehta

**Affiliations:** ^1^Division of Surveillance, Hazard Evaluations, and Field Studies, National Institute for Occupational Safety and Health, CDC; ^2^Robert H. Lurie Comprehensive Cancer Center, Northwestern University, Chicago, Illinois.

In 2014, CDC received a request from workers at a natural gas company in Illinois for a health hazard evaluation. The request concerned a perceived cluster of amyloidosis and multiple myeloma among workers. The company delivers natural gas to residential and business customers and employs approximately 1,300 persons. Employees are classified into three job groups: administrative, service, and distribution. Plasma cell dyscrasias, characterized by the monoclonal growth of plasma cells, include multiple myeloma, Waldenstrom macroglobulinemia (WM), monoclonal gammopathy of undetermined significance (MGUS), and amyloidosis. Using a standard approach (*1*), CDC investigated this suspected cluster. Investigators obtained information from the company’s two health insurance providers to identify current and former employees with these diagnoses from January 2008–January 2014 using *International Classification of Diseases, Ninth Revision* codes. Diagnoses were confirmed by contacting health care providers or reviewing medical records. Demographic and work information was obtained from the company.

Thirteen workers with confirmed plasma cell disorders were identified, including two active and 11 retired employees. Diagnoses included MGUS (five persons), myeloma (four), WM (three), and immunoglobulin light chain amyloidosis (one). All affected employees were men; eight were white, and five were black. The median age at diagnosis was 72 years (range = 38–90 years). Four employees received their diagnoses while they were active employees; nine diagnoses were made during retirement. Years of hire ranged from 1946 to 1995; years of retirement or termination ranged from 1982 to 2014. The median time worked at the company before diagnosis or retirement (whichever was earlier) was 31 years (range = 15–50 years). Job categories included distribution (five persons), service (five), and administrative (three). Work locations included five different shops and office locations. Each affected employee had one or more demographic risk factors for plasma cell dyscrasias, including male sex (myeloma, MGUS, and WM), older age (all diagnoses), and black race (myeloma and MGUS).

Company representatives estimated that 30,000–50,000 persons had worked for the company since 1946. It was not possible to calculate crude or adjusted incidence rates among employees because the cumulative number of company employees could not be determined. Therefore, statistical comparisons between employees and the general Illinois population were not possible and might not be appropriate. Also, disease or tumor rates are highly variable in small populations and rarely match the overall rate for a larger area such as an entire state. Nonetheless, available national information was used to crudely estimate rates.

Using published estimates for the lifetime risk for developing multiple myeloma (1 in 125),* four multiple myeloma cases did not appear unusual. Using the reported prevalence of MGUS (1%–3% in persons aged ≥50 years) (*2*), five MGUS cases also did not appear unusual. However, WM is rare, with an incidence of three cases per 1 million per year nationwide.^†^ Therefore, the occurrence of three cases among persons working for the same employer did appear unusual. They might be a coincidental occurrence, or the cases might represent exposures to an unproven causative agent.

No environmental or occupational exposures have been definitively established as causes for any plasma cell disorder. However, benzene, pesticides, coal dust, and organic solvent exposures have been associated with myeloma, WM, and MGUS (*3*–*5*). According to company representatives, it was unlikely that employees were exposed to these substances. Also, the three employees with WM worked in three different areas (one each in administrative, service, and distribution) and therefore likely had different exposures.

This investigation highlights the difficulty of elucidating whether clusters of plasma cell dyscrasias result from chance or if they have a common occupational or environmental cause. This difficulty is partly a consequence of the lack of occupation and industry information in most disease registries, including cancer registries. By disseminating information about clusters such as this one, more accurate reporting of usual (or longest held) occupation and industry data in medical records can be encouraged, so that surveillance systems and registries can be used to stimulate research on occupational causes of cancer.

## Footnotes

* https://seer.cancer.gov/statfacts/html/mulmy.html.

†
https://www.cancer.org/cancer/waldenstrommacroglobulinemia/detailedguide/waldenstrom-macroglobulinemia-key-statistics-w-m .
